# Midlife Work Disability and Illness Spells: New Data from the HRS 2019 Life History Survey

**DOI:** 10.1093/geroni/igaf122.3812

**Published:** 2025-12-31

**Authors:** Jessica Kelley, John Whesu, Yuanchang Zhao, Wenxuan Huang

**Affiliations:** Case Western Reserve University, Cleveland, Ohio, United States; Case Western Reserve University, Cleveland, Ohio, United States; Case Western Reserve University, Cleveland, Ohio, United States; Johns Hopkins University, Baltimore, Maryland, United States

## Abstract

Midlife is a critically important time in the life course. Adults in midlife are the intergenerational linkage between their (grand)parents and their (grand)children. Spells of ill health or disability can place pressures on the time, money, and energy that midlife individuals, potentially setting off a cascade of adverse circumstances that degrade later-life well-being. Adding the newly released data (2019) from the Health and Retirement Study Life History Survey to the previous two (2015, 2017), we construct joint sequences on 12,909 adults linking their health and work status between the ages of 20 and 63. Health sequences included spells of serious illness, injury, and disablement. Work sequences included work status (FT, PT, retired, not working, education), along with spells of non-work due to medical or disability reasons from age 20 to 63. Combining these sequences, we show that 78% of adults never had a serious illness or had to leave a job for medical reasons, while 17% experienced at least one serious illness that lasted more than a year, or left a job for medical reasons, or both. Another 4.5% were ill their entire lives or never worked due to disability. Median age of first serious illness is 41, and median age of leaving work for medical reasons is 50. We present these joint sequences, along with preliminary associations with later-life outcomes of depressive symptoms, loneliness, and wealth. We further discuss the value of combining work and health sequences in life history data for a fuller picture of midlife health.

